# Importance of cerebral angiography in the evaluation of delayed carotid stent thrombosis: a case report

**DOI:** 10.1186/s13256-024-04379-5

**Published:** 2024-02-22

**Authors:** Zaki Masoud, Juan Felipe Daza-Ovalle, Charles Esenwa

**Affiliations:** Department of Neurology/Albert Einstein College of Medicine, The Stern Stroke Center at Montefiore Health System, 3316 Rochambeau Ave, 4th Floor, Bronx, NY 10467 USA

**Keywords:** Carotid artery stenting, Digital subtraction angiography, Delayed carotid stent thrombosis, Dual antiplatelet therapy

## Abstract

**Background:**

While noninvasive imaging is typically used during the initial assessment of carotid artery disease, digital subtraction angiography remains the gold standard for evaluating carotid stent thrombosis and stenosis (Krawisz in *Cardiol Clin* 39:539–549, 2021). This case highlights the importance of digital subtraction angiography for assessing carotid artery stent patency in place of non-invasive imaging.

**Case presentation:**

We present a 61-year-old African American male patient with a history of right cervical internal carotid artery dissection that was treated with carotid artery stenting and endovascular thrombectomy, who developed recurrent right hemispheric infarcts related to delayed carotid stent thrombosis. Digital subtraction angiography found multiple filling defects consistent with extensive in-stent thrombosis not clearly observed with magnetic resonance angiography. Etiology was likely secondary to chronic antiplatelet noncompliance. Therefore, the patient was treated medically with a heparin drip, and dual antiplatelet therapy (dAPT) was restarted. At 1-month follow-up the patient did not report new motor or sensory deficits.

**Conclusion:**

In the setting of delayed carotid stent thrombosis secondary to antiplatelet noncompliance, digital subtraction angiography may play an essential diagnostic role for early identification and determination of the most appropriate treatment.

**Supplementary Information:**

The online version contains supplementary material available at 10.1186/s13256-024-04379-5.

## Introduction

Carotid stent thrombosis is an exceptionally rare and potentially fatal complication after carotid artery stenting (CAS), with limited reporting in the acute setting that usually manifests as a stroke secondary to thromboembolic mechanism [1]. This complication can be suspected with the worsening of neurologic deficit and a history of medication noncompliance. The scarcity of literature regarding carotid stent thrombosis is even more pronounced in the delayed setting. Furthermore, there is currently no established definition regarding the timeframe to distinguish delayed carotid stent thrombosis (dCSTh) from acute or subacute carotid stent thrombosis. Despite the acknowledged limitations of noninvasive imaging in the acute setting, there is uncertainty regarding diagnostic imaging recommendations for dCSTh. In this context, three additional factors should be considered: the time of thrombosis, severity of new neurologic deficit, and the extension of infarcted territory [2]. Consideration of these can aid in minimizing complication events, such as future embolic stroke or hemorrhagic transformation [2]. In addition to antiplatelet resistance [1, 2] or inadequate antiplatelet treatment [3], other possible risk factors for dCSTh are heparin resistance, diabetes mellitus [1], severe plaque protrusion [4], and early stent restenosis [5].

## Case presentation

**Fig. 1:**
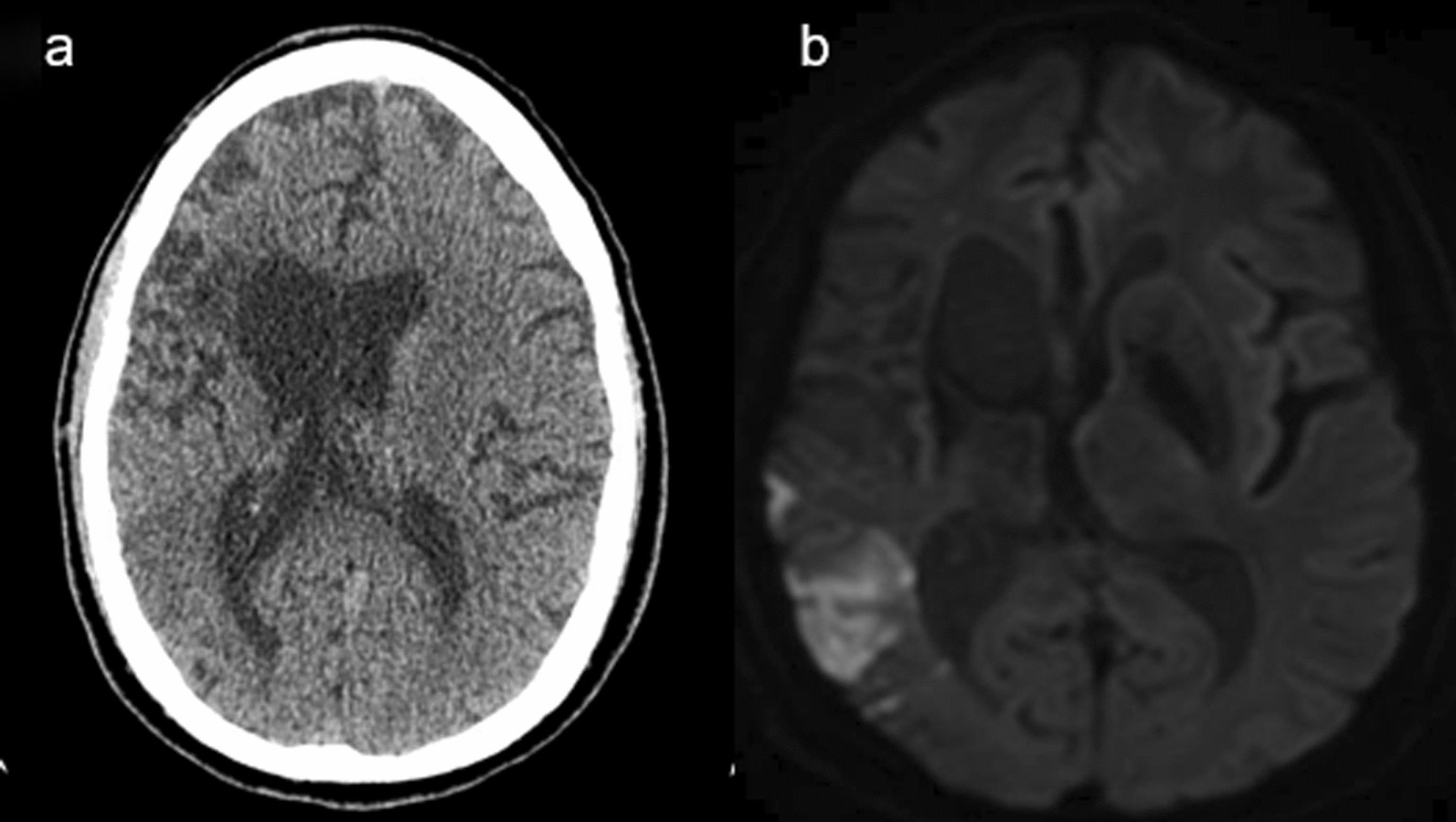
**a** Computerized tomography of the head without contrast showing chronic right middle cerebral artery (MCA) territory infarct including an area of evolving acute/subacute left temporal and parietal lobe ischemia. Shown better on** b** magnetic resonance imaging (MRI) showing acute right MCA territory infarct involving the right temporal lobe, right parietal lobe, and right precentral gyrus

**Fig. 2:**
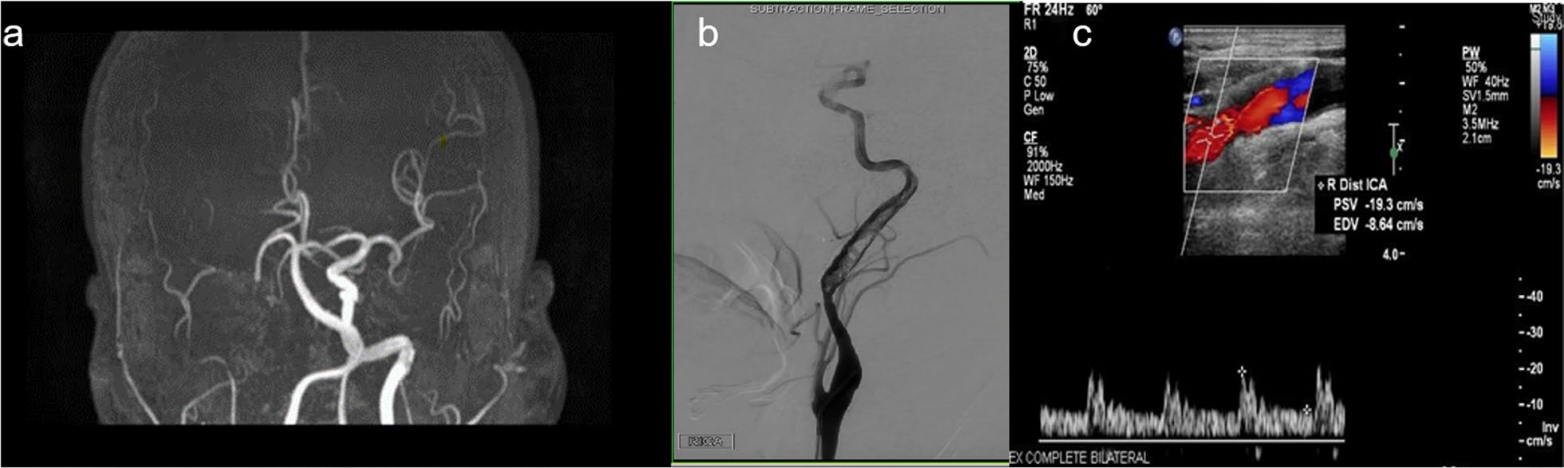
**Relevant neuroimaging showcasing key imaging findings**.** a** Right cervicocerebral angiogram with multiple filling defects at the level of the carotid stent just proximal to the petrous bone. Further filling defects extending up through the peak petrous and cavernous segments suggests thrombosis. There is antegrade flow into the middle and anterior cerebral arteries.** b** Absent flow signal in the right cervical internal carotid artery (ICA) system thought to be pseudo-occlusion.** c** Right spectral carotid Doppler analysis showing blunting of the arterial waveform consistent with nonhemodynamically significant stenosis of 1–49% in the distal internal carotid artery

A 61-year-old, right-handed African American man presented to our emergency department with acute worsening of chronic left hemiparesis. He had a history of right middle cerebral artery (MCA) M1 occlusion owing to right cervical internal carotid artery dissection that was treated with endovascular thrombectomy and emergent CAS with a 4 mm × 23 mm Enterprise stent 4 years prior. At the time, he was placed on dAPT and was recommended life-long aspirin; however, he was not fully compliant in the last year. On neurological examination, he was found to have left hemibody spasticity with left arm plegia. The left leg demonstrated partial weakness with drift at 5 seconds, with 3/5 strength and 5/5 strength on the right side. Magnetic resonance imaging and computerized tomography of the head revealed acute infarctions in the right MCA territory involving the right temporal lobe, right parietal lobe, and right precentral gyrus, abutting extensive chronic infarct in the right MCA territory (Fig.1a and b). Time-of-flight (TOF) MRA of the head and neck (without contrast) found loss of normal flow-related signal throughout the right carotid system and MCA (Fig. 2a), a finding consistent with cervical internal carotid artery occlusion. Digital subtraction angiography (DSA) was pursued to better characterize the lesion (Fig. 2b), which showed multiple filling defects at the stent level in the cervical and petrous right carotid segments, consistent with extensive dCSTh (as shown in Additional file 1: Video S1). There was no evidence of intracranial large vessel occlusion. Subsequently, the patient was treated medically with heparin drip because the patient was outside the window for thrombolysis, then dAPT was restarted when the patient was discharged. At 1-month follow-up, the patient denied worsening weakness or sensory problems, with good compliance of dAPT. On examination, the left arm had spasticity without movement and the left leg had drift, but it was maintained for 5 seconds with unstable gait and limited ambulation with a walker. He was transitioned from dAPT to combined therapy with aspirin plus apixaban.

## Discussion

CAS has become an important treatment approach for carotid artery stenosis in recent years [6]. However, dCSTh is a rare yet serious complication, with low-end estimates ranging from 2.7%, 2.6%, and 2.4% at 1, 2, and 3 years, respectively [7]. While noninvasive imaging techniques, such as MRA and Computerized Tomography Angiography, are typically used to detect carotid stent thrombosis and stenosis to avoid imaging procedural risks [8], DSA is crucial for assessing the patency of the carotid artery stent.

This case emphasizes the importance of DSA as the gold standard confirmatory test for carotid stent thrombosis and stenosis because it can identify discrepancies with noninvasive imaging [9] [such as ultrasound (Fig. 2c) or MRA]. Understanding the diagnostic discrepancy between DSA and other noninvasive imaging is crucial to defining whether medical treatment, open or an endovascular approach is needed. In addition, it is important to note that DSA provides information about collateral flow, atherosclerotic features, and severity of plaque morphology [8]. By emphasizing in the presence or absence of these radiologic features along the carotid arteries, DSA offers systematic assessment of atherosclerotic and stent morphology, defining its patency, plaque characteristics, collateral circulation, and grade of intracranial atherosclerotic disease [10].

A multicenter retrospective analysis from 2018 demonstrated that with reviewing the DSA, medical or surgical decisions changed in 107 of 243 cases (47%) in the setting of carotid stenosis [9]. Nonetheless, the use of noninvasive imaging has increased due to safety and the ability to obtain high resolution images of the carotid arteries [8]. Additionally, TOF MRA is an option in patients with gadolinium contrast contraindication but is less accurate in identifying moderate-to-severe carotid stenosis [8] or partial thrombosis when compared with DSA.

Furthermore, this case highlights the importance of continued life-long antiplatelet use. Despite the scarce available data around the etiology of carotid stent thrombosis, resistance to antiplatelet therapy or discontinuation are believed to be the main causes of stent thrombosis [2], as was inferred with our patient. Inherent or acquired thrombotic disorders like essential thrombocythemia or chronic atrial fibrillation, respectively, are other less common etiologies to be considered [2].

## Conclusion

dCSTh is an uncommon but serious CAS complication that should be considered in the setting of dAPT noncompliance or antiplatelet therapy resistance. Although noninvasive imaging methods are commonly employed to assess carotid artery disease, it is crucial to consistently consider DSA as it provides essential insights into stent patency, collateral blood flow, atherosclerotic characteristics, and the extent of plaque morphology [8].

### Supplementary Information

**Additional file 1: Video S1.** DSA shows multiple filling defects at the stent level in the cervical and petrous right carotid segments.

## Data Availability

No new data were created or analyzed during this study.

## Abbreviations

MCA: Middle cerebral artery
ICA: Internal carotid artery
CAS: Carotid artery stenting
dAPT: Dual antiplatelet therapy
TOF MRA: Time-of-flight magnetic resonance angiogram
dCSTh: Delayed carotid stent thrombosis
MRA: Magnetic resonance angiography
DSA: Digital subtraction angiography
